# Application of a novel disinvestment research design to the use of weekend allied health services on acute medical and surgical wards - randomised trial and economic evaluation protocol

**DOI:** 10.1186/1472-6963-14-S2-P53

**Published:** 2014-07-07

**Authors:** Terry Haines, Elizabeth Skinner, Deb Mitchell, Lisa O’Brien, Kelly Bowles, Donna Markham, Samantha Plumb, Timothy Chui, Kerry May, Romi Haas, David Lescai, Kathleen Philip, Fiona McDermott

**Affiliations:** 1 Physiotherapy Department, Monash University, Frankston, Victoria, Australia; 2 Allied Health Research Unit, Monash Health, Clayton, Victoria, Australia; 3 Physiotherapy Department, Western Health, Sunshine, Victoria, Australia; 4 Occupational Therapy Department, Monash University, Frankston, Victoria, Australia; 5 Allied Health, Monash Health, Clayton, Victoria, Australia; 6 Physiotherapy Department, Melbourne Health, Melbourne, Victoria, Australia; 7 Workforce, Leadership and Development Branch, Department of Health (Victoria), Melbourne, Victoria, Australia; 8 Social Work Department, Monash University, Caulfield, Victoria, Australia

## Background

Some currently provided health services have an absence of evidence for effectiveness, cost-effectiveness and/or safety. These are candidates for disinvestment. It is possible that such services would prove valuable if trials were to be conducted however, making disinvestment a clear risk. Provision of these services in the context of usual care is a considerable barrier to conducting a conventional trial of these interventions. Our team has recently developed a novel research approach to conduct a trial in this context [1]. In this paper, we describe the first application of this design.

Allied health services include those provided by a range of health professional groups. Weekend allied health services on acute medical or surgical wards are widely provided internationally but are inconsistent in their composition and focus. There is no direct evidence of efficacy for these weekend services, and higher rates of pay on the weekend make their likely cost-effectiveness questionable. This research examines the efficacy, cost-effectiveness and safety of disinvesting from weekend allied health services on acute medical or surgical wards.

## Materials and methods

This research consists of two multi-site stepped wedge cluster randomised trials (three hospitals and 17 wards). The first at each site is the novel, disinvestment, roll-in, stepped wedge, while the second is the conventional roll-out, stepped wedge. During the first trial, an additional ward loses its existing weekend allied health service each calendar month creating a seven stepped trial at two sites and a six step trial at one. This trial has a non-inferiority research paradigm with monthly safety checks and trial stopping rules to monitor patient safety and hospital flow outcomes. During the second trial, a new, stakeholder-driven model of weekend allied health service is introduced to an additional ward each calendar month.

The primary outcome measures in this trial are length of stay (mean length of stay, proportion of patients who stay longer than their expected length of stay), the proportion of patients who experience an adverse event (composite outcome), and the proportion of patients who are readmitted within 28 days of discharge. Random subsampling and qualitative methods are being employed to collect a range of secondary outcomes.

## Results and conclusions

This trial commenced in February 2014 and is due for completion across all sites in 2015.
